# Dietary pattern and nutritional assessment in a cohort of mothers identified by neonatal screening for cobalamin deficiency in offspring: an Italian single center experience

**DOI:** 10.3389/fnut.2025.1604336

**Published:** 2025-06-04

**Authors:** Martina Tosi, Veronica Maria Tagi, Alice Colombo, Alessandra Cecchini, Marianna Zobele, Chiara Montanari, Simona Ferraro, Alessandra Bosetti, Eleonora Bonaventura, Fabio Bruschi, Diego De Zan, Giulia Fiore, Cristina Cereda, Gianvincenzo Zuccotti, Elvira Verduci

**Affiliations:** ^1^Department of Pediatrics, Vittore Buzzi Children's Hospital, Milan, Italy; ^2^Department of Health Sciences, University of Milan, Milan, Italy; ^3^Department of Biomedical and Clinical Science, University of Milan, Milan, Italy; ^4^COALA (Center for Diagnosis and Treatment of Leukodystrophies), Unit of Pediatric Neurology, Vittore Buzzi Children’s Hospital, Milan, Italy; ^5^Unit of Pediatric Neurology, Vittore Buzzi Children’s Hospital, Milan, Italy; ^6^Center of Functional Genomics and Rare Diseases, Vittore Buzzi Children’s Hospital, Milan, Italy; ^7^Metabolic Diseases Unit, Department of Pediatrics, Vittore Buzzi Children's Hospital, Milan, Italy

**Keywords:** pregnancy, nutritional deficiencies, cobalamin, dietary pattern, vegetarian, food supplements

## Abstract

During pregnancy, nutrient requirements increase while deficiencies can significantly affect pregnancy outcomes. Deficiencies may result from inadequate dietary intake, impaired absorption, or restrictive diets. This study aimed to retrospectively assess the nutritional status and dietary intakes in a cohort of mothers whose newborns were identified with vitamin B12 deficiency of maternal origin through Newborn Screening. Between 2021 and 2024, 107 newborn-mother dyads with altered biomarkers of cobalamin metabolism were identified and referred to the Metabolic Disease Unit for further evaluation and treatment. Mothers underwent biochemical assessments and nutritional interviews regarding pregnancy diet history, and dietary intakes were quantified using a dedicated software (MetadietaVR). Most of the cohort (47%) was from Asia, with an average age of 32.5 years. Plasma vitamin B12 levels averaged 240 pg/ml. Mothers who reported taking vitamin B12 supplements had higher plasma levels compared to those who did not supplement with B12 (255.5 ± 113 vs. 231.2 ± 104 pg/ml). Dietary habits during pregnancy revealed that 71% of mothers were omnivorous (O), 16% followed a lacto-vegetarian (LV) diet, 12% a lacto-ovo-vegetarian (LOV) diet, and 1% a vegan (V) diet. Most mothers (90%) were taking supplements during pregnancy, with 70.7% taking folic acid, 68.7% iron and 15% vitamin B12. Among women who achieved adequate vitamin B12 intake through both diet and supplementation, 95% were omnivores while 5% followed a LOV diet. This study emphasizes the importance of addressing maternal nutritional needs from the pre-conception period, as dietary patterns may not adequately reflect micronutrient intake. Even omnivorous diets, if unbalanced, can result in insufficient nutrient intake, underlying the necessity of targeted nutritional support during pregnancy.

## 1 Introduction

Pregnancy is a critical period marked by rapid and significant physiological changes that heighten the body’s need for macro- and micronutrients to support maternal metabolism and fetal development (1). While these nutritional requirements can be met through a well-balanced diet, supplementation is often necessary to ensure an adequate intake of essential nutrients such as folic acid, which is vital in preventing neural tube defects (2). Dietary patterns play a crucial role in determining micronutrient intake during pregnancy, as different eating habits can affect nutrient adequacy and may predispose pregnant women to deficiencies. Research suggests that deficiencies in key nutrients such as iron, calcium, vitamin D, vitamin B12, folate, and docosahexaenoic acid (DHA) are common among pregnant women and may contribute to pregnancy complications and adverse neonatal outcomes (3–11). Iron deficiency, for instance, is the most widespread single-nutrient deficiency worldwide and has been linked to low birth weight and an increased risk of preterm delivery (12). Among various dietary patterns, vegetarian and vegan diets warrant special consideration during pregnancy, as they can lead to an inadequate intake of important micronutrients, including vitamin B12, iron, and omega-3 fatty acids (13, 14). Vitamin B12 deficiency is particularly concerning, as it is naturally found only in animal-derived foods, making supplementation or fortified foods necessary for people on plant-based diets (15). This vitamin plays a key role during pregnancy, as it is essential in fetal growth and neurodevelopment. Maternal vitamin B12 deficiency has been linked with an increased risk of fetal growth restriction, premature birth, and low birth weight (16). Additionally, newborns of mothers with B12 deficiency may present neuromuscular and developmental impairments, including hypotonia, lethargy, feeding difficulties, seizures, and electroencephalographic abnormalities, which in severe cases can lead to developmental delay or regression (17–19). Postnatally, B12 deficiency can manifest with megaloblastic anemia, hepatosplenomegaly, poor appetite and failure to thrive. Furthermore, recent studies suggest a potential association between B12 deficiency and non-communicable diseases through epigenetic regulation mechanisms (20). Maternal nutrition extends its influence beyond immediate pregnancy results, playing a significant role in long-term offspring health. The *Developmental Origins of Health and Disease* hypothesis suggests that nutritional deficiencies during pregnancy may have lasting effects on metabolic function and disease susceptibility in adulthood (21). Given the direct connection between maternal micronutrient intake and neonatal health, ensuring adequate B12 levels is crucial for optimal fetal development, particularly since maternal nutrition is linked to neonatal growth outcomes, even in women with a high body mass index (BMI) but no signs of overt malnutrition (22). The aim of our study is to retrospectively evaluate the nutritional status and dietary habits of a cohort of mothers identified through Newborn Screening (NBS) for cobalamin deficiency in offspring.

## 2 Materials and methods

From November 1st, 2021, to September 30th, 2024, the Regional Reference Laboratory for Newborn Screening for Lombardy Region (Italy) screened 199,526 newborns. Of these, 107 newborns exhibited a negative NBS while having altered biomarker levels suggestive for B12 deficiency of maternal origin, as indicated by increased levels of methylmalonic acid (MMA) and/or homocysteine (Hcy), along with normal levels of methylcitric acid and 3-hydroxypropionic acid on the first dried blood spot (DBS) sample. A second DBS sample was then collected from the birth center to confirm the initial findings. At the same time, maternal and neonatal serum and plasma samples were analyzed for B12 and Hcy concentrations. If results matched, a diagnosis of neonatal B12 deficiency secondary to a maternal condition was confirmed. All 107 mother-newborn pairs were referred to the Metabolic Disease Unit and Clinical Nutrition Service of Vittore Buzzi Children’s Hospital for further evaluation and treatment with hydroxycobalamin supplementation. This study was approved by the Local Ethics Committee LOMBARDIA 1 (protocol n #CET 132–2023 of December 6th, 2023). All procedures adhered to the guidelines of the Local Ethical Committee and were performed in accordance with the ethical standards of the 1964 Helsinki Declaration. Informed consent for the treatment of anonymized data was obtained from a parent or a legal guardian. Data was reported and stored on the REDCap platform.

### 2.1 Hematological and biochemical assessments

Following referral to the Regional Reference Laboratory for Neonatal Screening, a first multidisciplinary evaluation was scheduled between 20 and 30 days of age of infants. During this first visit, mothers underwent a thorough hematological and biochemical assessment, including a complete blood count with peripheral blood smear. Biochemical testing involved measuring plasma levels of vitamin B12, homocysteine, and folic acid to assess micronutrient status. Folic acid and total B12 were measured using Elettrochemiluminescence (ECLIA) Roche Cobas, and homocysteine levels were analyzed by Chemiluminescent Microparticles Immunoassay (CMIA) Abbott Alinity. Iron status was assessed by measuring serum iron, ferritin, and transferrin levels. Nutritional status was further evaluated by testing prealbumin, albumin, and total protein levels. Celiac disease was screened using anti-tissue transglutaminase IgA anti-bodies (anti-TTG IgA reflex test), while anti-parietal cell antibodies were assessed to rule out atrophic gastritis. Moreover, urinary MMA levels were measured as an indirect index of vitamin B12 deficiency. Urinary MMA concentrations were measured on 2 ml urine samples by a LC–MS/MS system (Sciex Triple Quad 6,500), together with the internal quality control (ERNDIM Internal Quality Control System (IQCS) Special Assays in Urine). The concentration of urinary creatinine was determined, and urinary MMA was expressed as mM/M creatinine. All these assessments were conducted following established internal clinical procedures, and the results were compared to reference values.

### 2.2 Nutritional assessment

During the initial evaluation for infants, mothers’ dietary history during pregnancy was retrospectively collected by registered dietitians to identify potential nutritional factors contributing to vitamin B12 deficiency. This assessment included a retrospective examination of the mothers’ pre-pregnancy weight, total weight gain throughout pregnancy, and the post-pregnancy weight, which was measured on the day of the visit. Considering the entire pregnancy, the dietitians meticulously documented dietary intakes, including main meals (breakfast, lunch, and dinner) and snacks consumed throughout the day and at bedtime. A photographic food atlas (*Atlante Fotografico delle Porzioni degli Alimenti* developed by the Istituto Scotti Bassani) was employed to estimate the portions size, allowing for an accurate determination of the actual food intake. These dietary assessments were conducted by analyzing changes in eating habits, food consumption frequencies across the trimesters, with particular emphasis on animal-source foods. Portion sizes, consumption frequencies, cooking methods and the locations of meals were recorded. The evaluation also considered factors such as nausea or vomiting, which could have influenced food intake, ensuring a comprehensive understanding of the mothers’ nutritional status during pregnancy. Each food item, including portion size and frequency of consumption, was entered into a dedicated software (MetadietaVR; METEDAsrl, via S. Pellico 4, San Benedetto del Tronto, AP, Italy), designed to calculate a weighted average of energy, macronutrients, and micronutrients intake, providing a detailed and accurate nutritional analysis. The mean intake levels of macro and micronutrients were then compared to the Dietary Reference Value (DRVs) established by the European Food Safety Authority (EFSA) (23) for pregnant women. The specific reference value considered for each nutrient was chosen depending on the available data and included the average requirement (AR) for calcium and iron, population reference intake (PRI) for calcium and iron, and adequate intake (AI) for magnesium, phosphorus and potassium. Zinc reference values were estimated by adding AR and PRI for pregnancy (respectively, +1.3 mg/day and +1.6 mg/day) to the AR and PRI for adult women. Mothers were then classified into five dietary groups based on their dietary habits: omnivores (O) consuming all animal-derived protein sources, lacto-ovo-vegetarian (LOV) excluding meat, fish and poultry but including eggs and dairy products, lacto-vegetarian (LV) for those eating only dairy products, ovo-vegetarian (OV) eating only eggs, and vegan (V) for those excluding all animal-derived protein sources. Additionally, the use of vitamin and mineral supplements during pregnancy was documented, with questions focusing on the timing and dosages taken.

### 2.3 Covariables

Different covariables were also collected. For the neonate, the type of infant feeding was documented, with categories defined as: exclusively breastfed infants (receiving only breast milk), bottle-fed infants (receiving only infant formula) and mixed feeding (receiving both breast milk and infant formula). Demographic data for mothers, including maternal age and nationality, were gathered post-pregnancy. Nationality was categorized as follows: Italy, and other continents, Western Europe, Eastern Europe, Asia, Africa, North America, South America and Oceania.

### 2.4 Statistical analysis

All continuous variables are expressed as mean ± standard deviation (SD), while categorical variables are presented as percentage (%). The normality of continuous variables was assessed using the Kolmogrov-Smirnov and Shapiro–Wilk tests. To compare the mean plasma values of hemoglobin (Hb), folate and vitamin B12 across different diet groups, the Kruskal Wallis test was applied. Statistical comparisons were made only among O, LOV and LV, excluding the OV and V groups due to insufficient sample size in both categories. The Chi-square test (Χ2 test) was applied to evaluate whether specific diet adherence (O, LOV, LV expressed as %) was significantly associated with low ferritin levels (defined 0 for values ≥30 μg/L; 1 for values <30 μg/L), or insufficient hemoglobin values (defined 0 for values ≥12 g/dl; 1 for values <12 g/dl). Differences in plasma vitamin B12, hemoglobin and mean corpuscular volume (MCV) between dietary vitamin B12-supplemented and non-supplemented groups were assessed using the Unpaired t test for normally distributed variables and the Mann–Whitney tests, for not-normally distributed ones. The correlation between the presence of anti-gastric parietal cell antibody (APCA) and vitamin B12 was analyzed using the Spearman test. Statistical analysis was performed using GraphPad Prism (version 9.0.0 for Windows, GraphPad Software, Boston, Massachusetts USA, www.graphpad.com). A *p* < 0.05 was considered statically significant.

## 3 Results

### 3.1 Study population

The study included 107 mother-newborn pairs with vitamin B12 deficiency. Of these, 31% (n.33) of mothers were from Italy, while the remaining came from different ethnic backgrounds: 47% (n.50) were from Asia, 9% (n.10) from Africa, 8% (n.9) from Eastern Europe and 5% (n.5) from South America. The average age of the mothers was 32.5 years, with ages ranging from 15 to 46 years. A small percentage (10%, n.11) were teenagers or young adults (aged < 25 years), 22% (n.24) were between 26 and 30 years, while most (68%, n.72) were > 31 years. Regarding infant feeding, 62% (n.66) were exclusively breastfed, 10% (n.11) were bottle fed and 28% (n.30) received a combination of breast milk and infant formula.

### 3.2 Biochemical and clinical assessment in mothers and offspring

Mothers’ mean plasma values revealed: B12 240 pg/ml (SD ± 116) and Hcy 14.2 μmol/L (SD ± 6.7). In addition, 33% (n.35) of them had Hb levels below 12 g/dl. Regarding MCV, 16% (n.17) had values below 80 fl, and 5.5% (n.6) above 100 fl. Transferrin saturation was lower than 20% in 26% (n.28) of women. Additionally, 10% (n.11) of the total sample tested positive for APCA, and 11% (n.12) experienced hyperemesis gravidarum. No cases of coeliac disease were reported. Table 1 summarizes the key plasmatic and urinary results related to cobalamin metabolism obtained from both mothers and newborns prior to hydroxycobalamin administration. Newborns at first admission, before hydroxycobalamin administration, presented the following mean values: plasma vitamin B12 175.76 pg/ml (SD ± 75.32), plasma homocysteine 18.26 μmol/L (SD ± 8.75) (normal values <10 μmol/L), plasma Methylmalonic Acid (MMA) 3.50 μmol/L (SD ± 2.73) (normal values 0–4 μmol/L), and urinary MMA 54.96 μmol/L (SD ± 55.56) (normal values 0–2 μmol/L). To date, 71% (n.76) of children have reached the age of 12 months. No significant abnormalities in growth or neurological objective examination at neonatal age, at 6 and 12 months were observed. The assessment of psychomotor development at 24 months is in progress. Supplementary Table 1 shows the reference values for the hematological and biochemical parameters assessed.

**Table 1:** Plasma and urinary findings related to vitamin B12 metabolism at the first visit.

Mean plasma values	Mothers	Newborns
B12 (pg/ml)	240 (SD ± 116)	175.76 (SD ± 75.32)
Hcy (μmol/L)	14.2 (SD ± 6.7)	18.26 (SD ± 8.75)
MMA (μmol/L)	Not tested	3.50 (SD ± 2.73)
Mean urinary values		
MMA (mM/M creatinine)	Not tested	54.96 (SD ± 55.56)

### 3.3 Nutritional and dietetic assessment

Considering body weight, the average pre-pregnancy BMI was 25 kg/m2, with a range from 15.2 kg/m2 to 41.1 kg/m2. Most women (59%, n.63) were in the normal weight range (BMI 18.5–24.9 kg/m2), 35% (n.37) were either overweight or obese (BMI > 25 kg/m2), while a minority (6%, n.7) were underweight (BMI < 18.5 kg/m2). The average weight gain during pregnancy was 8.6 kg across all mothers, with variations by BMI category: 10.1 kg for underweight, 10.5 kg for normal weight, and 5.5 kg for overweight/obese mothers. Regarding dietary patterns during pregnancy, most mothers (71%, n.76) followed an O diet, 16% (n.17) adhered to an LV diet, and 12% (n.13) followed a LOV diet. Only 1% (n.1) followed a V diet. LOV, LV and V patterns were mostly followed by mothers of Asian descent. In particular, among Asian mothers, 48% (n.24) were omnivores, 20% (n.10) followed an LOV diet, 30% (n.15) adhered to an LV diet, and 2% (n.1) followed a vegan diet. Supplementary Table 2 presents the characteristics of the subjects, categorized by their B12 supplementation status, including also includes the analysis of the dietary patterns followed during pregnancy.

### 3.4 Supplementation during pregnancy

Most mothers (90%, n.96) were taking mineral or vitamin supplements during pregnancy. Of these, 70.7% (n.68) supplemented folic acid, 68.7% (n.66) iron (either alone or as part of a multivitamin), but only 15% (n.16) supplemented vitamin B12, always as part of a multi-vitamin, and the supplementation was reported to have been taken daily throughout the entire pregnancy. Notably, only 14% (n.13) supplemented both folate and vitamin B12. A small group (10%, n.10) did not take any supplement during pregnancy. Details about supplementation according to dietary patterns are shown in Supplementary Table 3. Among mothers who were supplemented with vitamin B12 during pregnancy, mean plasma vitamin B12 level was 253.5 ± 114 pg/ml. This was not significantly different from the plasma B12 levels in the non-supplemented group (231.2 ± 105, *p* = 0.54). Similarly, there were no significant differences between the supplemented and non-supplemented groups in terms of hemoglobin (12.3 ± 1.07 vs. 12.8 ± 1.1) and MCV (85.6 ± 7.4 vs. 87.7 ± 7.5).

### 3.5 Dietary intake compared with EFSA DRV

The intake of minerals (calcium, iron, magnesium, phosphorus, potassium, zinc) and vitamins (cobalamin, folate, vitamin D) was evaluated for all 107 mothers to determine whether it met the DRVs set by the EFSA. Table 2 shows the dietary intake of micronutrients during pregnancy, compared to EFSA DRVs, along with the percentage of mothers who achieved this intake, regardless of the dietary pattern followed during pregnancy. Several micronutrients were frequently consumed below EFSA DRVs. Vitamin D, folate, calcium (both AR and PRI), and iron PRI showed the most critical deficiencies, with nearly all participants failing to meet recommended levels. Magnesium, potassium, and vitamin B12 intake were also low in the majority of mothers. In contrast, phosphorus intake was generally adequate. When including supplementation, 21% (n.22) of women met the EFSA AI for vitamin B12, a 10% improvement compared to diet alone. Supplementary Table 4 shows the dietary intake of micronutrients and the percentage of mothers who achieved EFSA DRVs intake according to the vitamin B12 supplementation during pregnancy.

**Table 2:** Micronutrients dietary intake during pregnancy compared to EFSA DRVs for pregnancy.

Micronutrient	Mean dietary intake (min-max)	EFSA DRVs	% achieving EFSA DRVs
Calcium (mg/day)	675.7 (176–1708.5)	AR (18–24 years old): 860 AR (≥25 years old): 750 PRI (18–24 years old): 1,000 PRI (≥25 years old): 900	AR: 1% (n.1) AR: 36.4% (n.39) PRI: 0% PRI: 16.8% (n.18)
Iron (mg/day)	9.5 (2.4–29)	AR: 7 PRI: 16	AR: 78.5% (n.84) PRI: 2% (n.2)
Magnesium (mg/day)	210.4 (88.4–561.3)	AI: 300	7.4% (n.8)
Phosphorus (mg/day)	1034.6 (316.5–2183.5)	AI: 550	95.3% (n.102)
Potassium (mg/day)	2623.5 (1147.4–7356.7)	AI: 3500	14% (n.15)
Zinc (mg/day)	8.5 (3.2–19.6)	Range AR: 7.5–11.5 Range PRI: 9.1–14.3	AR: 48.5%^*^ (n.52) PRI: 35.5%^**^ (n.38)
Vitamin B12 (mcg/day)	2.6 (0–7)	AI: 4.5	11.2% (n.12)
Vitamin D (mcg/day)	1.5 (0.03–7.9)	AI: 15	0%
Folate (mcg/day)	306.7 (106.2–609)	AI: 600	<1% (n.1)

Average Requirement (AR), Population Reference Intake (PRI), Adequate Intake (AI).

* For Zinc AR: 14% (n.15) of women exceed the intake of 11.5 mg/day.

** For Zinc PRI: 3.7% (n.4) of women exceed the intake of 14.3 mg/day.

### 3.6 Dietary intake according to dietary pattern

**Figure 1:**
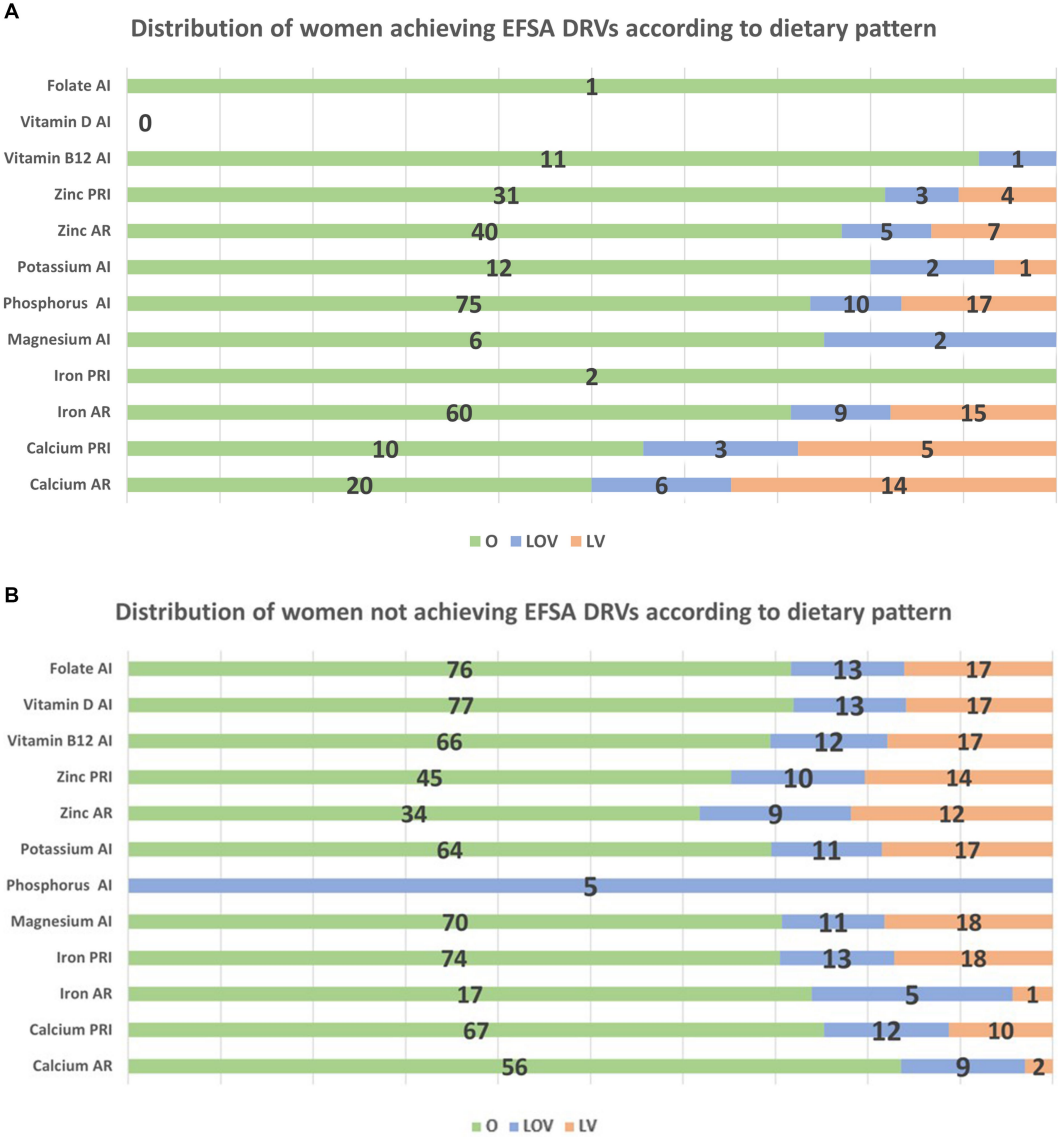
**(a)** Distribution of women achieving DRVs according to dietary pattern. *For Zinc, only lower level of the range 7.5–11.5 mg/day for AR and 9.1–14.3 mg/day were considered. Among women exceeding the zinc intake of 11.5 mg/day, they were 100% O, while among those exceeding 14.3 mg/day, 25% were LOV. **(b)** Distribution of women not achieving DRVs according to dietary pattern.

For women who met the EFSA DRVs, the dietary patterns associated with each micronutrient were analyzed. Figure 1a illustrates the distribution of women achieving DRVs according to dietary pattern (O, LOV and LV), considering only dietary intake, while Figure 1b shows the distribution of women not achieving DRVs, always according to dietary pattern. The V diet was excluded from the analysis due to its low representation in the sample. Interestingly, Figure 1a shows that only one mother achieved the DRV for folate through diet and she was omnivorous. Regarding vitamin B12, 91% of the mothers who consumed sufficient quantities to meet the DRVs were omnivorous, while 9% were LOV. In contrast, in Figure 1b, it can be observed that, for mothers who did not meet the DRV for vitamin B12, 69% were omnivorous, 13% were LOV, and 18% were LV. Similarly, all the data presented in the figures can be interpreted in this way. Regarding B12 and folate supplementation occurrence during pregnancy, of those mothers who met B12 AI, 95% followed an omnivorous diet while 5% a LOV diet. As for folate AI through supplementations, 78% were omnivores, 10% LOV and 12% LV. The mother following a V diet did not meet any of the DRVs through diet alone.

### 3.7 Association between diet type and biochemical parameters

No significant differences were observed in the mean plasma levels of hemoglobin, folate, and vitamin B12 among O, LOV, and LV. Similarly, the Chi-square test revealed no significant association between specific dietary adherence and low ferritin (40.7% O, 20% LOV, 26% LV; *p* = 0.33) or insufficient hemoglobin levels (24.1% O, 29.4% LOV, 23.8% LV; *p* = 0.89). Furthermore, no correlation was found between the presence of APCA and plasma vitamin B12 levels in the cohort of enrolled mothers (Spearman coefficient r = 0.0019, *p* = 0.98).

### 3.8 Causes of low blood vitamin B12 levels

The majority of low vitamin B12 blood levels were attributed to isolated low dietary intake of vitamin B12 (74%, n.79). Other contributing factors included hyperemesis gravidarum (10%, n.11), positive APCA (8%, n.9), Methylenetetrahydrofolate Reductase (MTHFR) mutation (4%, n.4), obesity (3%, n.3), and previous bariatric surgery (1%, n.1).

## 4 Discussion

In our study, we found that 74% of women with vitamin B12 deficiency had an isolated low dietary intake of this essential vitamin. According to EFSA (23), the adequate daily intake of vitamin B12 for adults is 4 mcg/day, but for pregnant women it rises slightly to 4.5 mcg/day. Our findings revealed that only 21% of women who supplemented vitamin B12 reached the recommended intake, while just 11% of women relying solely on their diet met the requirements. These findings align with existing literature, where the most common causes of poor cobalamin status are low dietary intake and malabsorption (24). Since vitamin B12 is mostly found in animal-derived foods, such as meat and fish, individuals following vegan or vegetarian diets are at greater risk of insufficient intake, especially during pregnancy, where the demand for B12 increases. This can increase the risk of deficiency among pregnant women following these dietary patterns (25). Interestingly, our study found that nearly half of the mothers with a cobalamin deficiency in our cohort were from Asia, particularly southern India, where low consumption of animal-based foods is common for cultural-religious reasons (26). While women following an omnivorous diet were more likely to meet their vitamin B12 intake requirements, significant variability still exists, especially among those who infrequently consume meat or fish, and no significant differences were observed in plasma vitamin B12 levels between omnivores, lacto-ovo-vegetarians, and lacto-vegetarian. This suggests that even omnivorous women are at risk of insufficient B12 intake, especially during pregnancy when nausea and dysgeusia can impact food choices and reduce the consumption of animal-derived proteins. Furthermore, in accordance with other studies (27), also mothers who supplemented with oral cobalamin exhibited low plasma B12 and high homocysteine serum levels, pointing out the importance of monitoring both direct and indirect markers of B12 status during pregnancy and breastfeeding. This underscores the importance of regular nutritional assessment, as even women on omnivorous diets with low meat or fish consumption may experience inadequate B12 intake during pregnancy. Our findings also revealed that dietary intake alone often fails to meet the requirements for vitamin D and folate during pregnancy. This is particularly noteworthy as these nutrients are crucial during pregnancy for both maternal health and fetal development (28). Vitamin D deficiency has been linked to adverse pregnancy outcomes such as gestational diabetes, preeclampsia, and low birth weight (29). Similarly, adequate folate intake is essential for preventing neural tube defects in the fetus (30). Despite dietary shortfalls, it is encouraging to note that a substantial proportion of women (70.7%) were taking folate supplements during pregnancy, reflecting widespread public health claims about its importance. Regarding mineral intake, while omnivorous women are more likely to meet the EFSA PRI and AR for iron and zinc during pregnancy, there remains a significant portion of omnivores who do not achieve these nutritional goals. This shortfall often results from unbalanced diets that do not fully address nutritional needs, highlighting that merely consuming animal products does not guarantee adequate mineral intake. The literature indeed supports our findings by documenting cases of iron deficiency even among omnivores (31). Additionally, it is noteworthy that the calcium needs are met by LOV and LV dietary patterns. These diets, which include dairy products, provide a valuable source of calcium, crucial for both maternal and fetal bone health. The study found that O women took more supplements during pregnancy compared to LOV and LV woman. This difference may be attributable to differences in health literacy and cultural background, given that many of the LOV and LV women were from Asian countries where vegetarian dietary practices are shaped by cultural tradition. Nevertheless, across all dietary pattern groups, nutritional deficiencies and insufficient supplement intake, including folic acid, were observed. Considering mineral deficiencies in pregnancy, Cetin et al. (32) emphasized that inadequate maternal micronutrient status is common even in high-income countries, underlining the need for urgent education in nutrition for healthcare professionals. This would enable tailored counseling based on regional dietary habits and specific conditions, ultimately improving outcomes for both infants and adults. The International Expert Consensus on Micronutrient Supplement Use (33) highlights established guidelines for some micronutrients but reveals significant gaps in recommendations for others like vitamin B12. According to our findings, this emphasizes the need to implement preventive strategies, including nutritional investigations and appropriate dietary interventions with adequate supplementation during pregnancy. It is essential to consider targeted supplementation based on individual dietary patterns, rather than providing multivitamin supplements containing all minerals and vitamins. However, micronutrient deficiencies are not solely determined by inadequate intake or provision; they can also result from impaired absorption, altered utilization, or increased losses (34). This is also elucidated in our study in cases of mothers with adequate intake but with relevant underlying factors including APCA, hyperemesis gravidarum, and bariatric surgery.

Offspring of women with B12 deficiency may manifest neurodevelopmental delay and neurological signs such as hypotonia, lethargy, feeding difficulties, seizures, and electroencephalographic abnormalities (17–19). Furthermore, failure to thrive during infancy has been described in literature (35). In our cohort of newborns, early supplemented with hydroxocobalamin, no significant abnormalities in growth or neurological objective examination were documented at neonatal age, at 6 and 12 months.

This study allowed for the identification of additional nutritional risks, based on a sample of women referred for vitamin B12 deficiency in offspring. It underscores that, in addition to the risks identified through screening, other nutritional deficiencies may coexist in the mothers, potentially leading to significant health implications for the offspring. Furthermore, it was observed that many of these women overlook the importance of nutrition, failing to adhere to appropriate supplementation for pregnancy or to adjust their supplementation according to their dietary patterns, often due to limited health and nutritional literacy. Lastly, it is emphasized that not all women received adequate prenatal support, during which appropriate supplementation could have been provided. While this study has some limitations, including a limited sample size and the absence of a control group, our findings align with previous studies showing a high prevalence of cobalamin deficiency during pregnancy (36). Furthermore, mothers were not monitored during pregnancy and the retrospective collection of dietary history relies on memory; therefore, the derived results should be considered approximations, reflecting the best available recall of dietary habits during pregnancy. To minimize recall bias, a portion size photographic atlas was used and dietitians received training to ensure a consistent approach. The retrospective design may be subject to recall bias, particularly in relation to self-reported supplementation practices. Consequently, the apparent lack of benefit from B12 supplementation during pregnancy should be interpreted with caution, as inaccurate reporting may have influenced the findings. Lastly, the patients may not be representative of the general population because they were identified specifically for having a deficiency detected by NBS. This could limit the generalizability of the study findings.

### 4.1 Open challenges in the diagnosis and treatment of cobalamin deficiency

Diagnosing vitamin B12 deficiency is challenging, as total serum B12 levels may not always reflect true deficiency. Several factors, including inter-laboratory variability and individual daily fluctuations, may interfere with an accurate measurement. Additionally, in individuals who are orally supplemented with vitamin B12, serum B12 concentrations may result within normal values even though deficiency symptoms persist (27). To overcome this issue, many experts recommend measuring at least one indirect marker of cobalamin deficiency, namely MMA and Hcy (37). However, the reliability of homocysteine and MMA concentrations as diagnostic tools for vitamin B12 deficiency remains a topic of debate among researchers. It is important to emphasize that elevated levels of homocysteine and MMA should not be used as first-line tests for diagnosing B12 deficiency. Rather, an increase in these markers above established cut-off values can help in identifying individuals who may require supplementation, particularly when serum B12 levels fall within the “grey zone” (traditionally defined as 100–300 ng/L) (38). More recently, this reference range has been updated to 180–350 ng/L, according to the UK National Institute for Clinical Excellence (NICE) clinical practice guidelines (CPG). Individuals with B12 serum levels within this range may still have a deficiency if they exhibit specific clinical signs and symptoms or if their serum MMA levels are elevated (39). Early screening for vitamin B12 deficiency, particularly during the preconception period or early pregnancy, could indeed allow for timely intervention and prevent long-term complications. Furthermore, preventing cobalamin deficiency in newborns, particularly when it is of maternal origin but not detected by neonatal screening, could significantly reduce the long-term public health costs associated with managing these cases (i.e., intramuscular injections and repeated blood samples). Lastly, it is well recognized that the measurement of holotranscobalamin is a more accurate marker of early B12 deficiency than total B12, particularly in pregnant women and newborns (40). During pregnancy, total B12 levels may appear falsely low due to reduced circulating haptocorrin and hemodilution (40). In newborns, high concentrations of unsaturated haptocorrin in human milk can also affect results (41). Experts recommend holotranscobalamin testing as it detects the active B12 fraction available to cells and is less prone to interference, yet most laboratories still rely on total B12 testing (38). A recent study from our working group (42) examined the clinical management and associated costs of treating newborns with B12 deficiency in Italy. The study showed that the cost of managing a newborn with vitamin B12 deficiency amounts to €3,578.6 (€3,875.75 for preterm infants), with €3,253.16 and €3,550.31, respectively, attributed to the Regional Health System, and €325.44 to caregivers. Moreover, the study projected an expected increase in annual costs from €207,735 to €428,803, highlighting that managing this condition has a significant burden on caregivers, involving financial costs but also considerable stress and logistical demands.

## 5 Conclusion

Based on the findings of our study, results showed that vitamin B12 deficiency in newborns was primarily associated with inadequate maternal intake during pregnancy in our study. Women following omnivorous diets were more likely to meet their EFSA DRVs compared to those following LOV and LV diets, but also within the omnivorous group some women may also be at risk for insufficient intake of micronutrients during pregnancy, highlighting the importance of nutritional assessment. Giving the significant risks associated with untreated vitamin B12 deficiency, raise awareness among healthcare professionals (i.e., general practitioners, gynecologists and obstetricians) about the importance of dietary assessment, especially for those on vegetarian or vegan diets, is crucial. This supports the need for a multidisciplinary management approach, which is currently not standard practice, and calls for early preventive strategies, ideally starting in the peri-conception period. Nutritional counseling and personalized supplementation are key to preventing deficiency and ensuring optimal health for both mothers and their offspring. Expanded newborn screening can also help in detecting vitamin B12 deficiency early, thus providing a window of opportunity for timely treatment. Addressing maternal nutritional needs from the pre-conception period is essential for promoting the long-term health of future generations.

## Data Availability

The raw data supporting the conclusions of this article will be made available by the authors, without undue reservation.
