# Supplementary material for: Dietary pattern and nutritional assessment in a cohort of mothers identified by neonatal screening for cobalamin deficiency in offspring: an Italian single center experience
Source: Front Nutr. 2025 Jun 4;12:1604336. doi: 10.3389/fnut.2025.1604336 (PMC12173883; doi:10.3389/fnut.2025.1604336)
Supplement: Supplementary file 1 [file Table_1.doc]

**Supplementary Table 1.** Reference values for hematological and biochemical parameters assessed.

| Hematological or biochemical parameter | Reference values (unit of measure) |
| --- | --- |
| Erythrocytes | 4.13-5-15 (x10^9/L) |
| Hemoglobin | 12.5-15.5 (g/dl) |
| Mean Corpuscular Volume | 81.8-95.3 (fL) |
| Hematocrit | 0.38-0.46 (L/L) |
| White blood cells | 4.19-9.35 (x10^9/L) |
| Lymphocytes | 1.13-3-37 (x10^9/L) |
| Neutrophils | 1.91-6.23 (x10^9/L) |
| Platelets | 169-359 (x10^9/L) |
| Vitamin B12 | >400 (ng/L) |
| Homocysteine | ≤10 (µmol/L) |
| Folic acid | >4 (µg/L) |
| Iron | 50-170 (µg/dL) |
| Ferritin | >30 (µg/L) |
| Transferrin | 2.0-3.6 (g/L) |
| Prealbumin | 0.2-0.4 (g/L) |
| Albumin | 35-50 (g/L) |
| Total proteins | 65-85 (g/L) |
| anti-tissue transglutaminase IgA anti-bodies | <7 (U/mL) |
| IgA | 0.70-4.00 (g/L) |
| Anti-parietal cell antibodies | Negative |
| Urinary MMA | 0.00-2.00 (mM/M Creat.ur.) |
| Urinary creatinine | 60-180 (mg/dl) |

**Supplementary Table 2.** Characteristics of the subjects included in the study, divided by their B12 supplementation status.

|  | B12-supplemented group | B12-non supplemented group |
| --- | --- | --- |
| N° of subjects | 16 | 91 |
| Mean age | 32.7 | 32.5 |
| Ethnicity | | |
| Italy | 7 | 26 |
| Asia | 4 | 46 |
| Africa | 3 | 7 |
| South America | 1 | 4 |
| Eastern Europe | 1 | 8 |
| Diet | | |
| O | 14 | 62 |
| LV | 2 | 15 |
| LOV | 0 | 13 |
| V | 0 | 1 |

**Supplementary Table 3.** Supplementation during pregnancy according to O, LOV, LV and V dietary patterns. Minerals and vitamins are considered single supplements. All the multivitamins contain folic acid.

| Dietary pattern | Supplementation during pregnancy | Range of the daily amount of dietary intake | Range of the daily amount of supplementation | Range of the daily total amount of dietary intake and supplementation |
| --- | --- | --- | --- | --- |
| O | Folic acid 68.6%  Iron 51.4%  Calcium 2.8%  Magnesium 2.8%  Vitamin D 7.1%  Multivitamin 21.4%  None 11.4% | (134**.**7-501**.**1 mcg)  (2**.**42-16**.**6 mg)  (388**.**6-1011**.**2 mg)  (120**.**9-561**.**3 mg)  (0**.**2-4**.**4 mcg)  ---  --- | (300-800 mcg)  (30-100 mg)  (140-250 mg)  (60-400 mg)  (13-50 mcg)  ---  --- | (434**.**7-1201**.**1 mcg)  (32**.**42-146**.**6 mg)  (528-1151**.**2 mg)  (180**.**9-961**.**3 mg)  (13**.**2-53**.**2 mcg)  ---  --- |
| LOV | Folic acid 75%  Iron 75%  Calcium 8.3%  Magnesium 8.3%  None 8.3% | (236.5-505.3 mcg)  (4.9-15.5 mg)  (192.9 mg)  (95.9 mg)  --- | (400 mcg)  (30-80 mg)  (140 mg)  (400 mg) | (636.5-905.3 mcg)  (34.9-97.5 mg)  (332.9 mg)  (495.9 mg) |
| LV | Folic acid 50%  Iron 75%  Calcium 6.3%  Vitamin D 6.3%  Multivitamin 6.3% | (172.3-395.8 mcg)  (3.7-10.6 mg)  (819.16 mg)  (0.42 mcg)  --- | (400 mcg)  (30-80 mg)  (250 mg)  (15 mcg)  --- | (572.3-795.8 mcg)  (33.7-85.6 mg)  (1069.16 mg)  (15.42 mcg)  --- |
| V | None 100% | **---** | **---** | **---** |

*Multivitamins were not calculated because of the variability of nutrients included.

**Supplementary Table 4.** Micronutrients dietary intake during pregnancy compared to EFSA DRVs for pregnancy. Average Requirement (AR), Population Reference Intake (PRI), Adequate Intake (AI) according to vitamin B12 supplementation during pregnancy.

| Micronutrient | Mean dietary intake  (min-max) | | EFSA DRVs | % achieving  EFSA DRVs in each group | |
| --- | --- | --- | --- | --- | --- |
|  | **B12 suppl.** | **No B12 suppl.** |  | **B12 suppl.** | **No B12 suppl.** |
| Calcium (mg/day) | 644 (388.6-1011.2) | 681.5 (176-1708.2) | AR (18-24 y): 860  AR (≥25 y): 750 | 40% | 36.9% |
|  |  |  | PRI (18-24 y): 1000  PRI (≥25 y): 900 | 13.3% | 21.4% |
| Iron (mg/day) | 9.3 (5.3-13.5) | 9.5 (2.4-29) | AR: 7 | 73% | 79.7% |
|  |  |  | PRI: 16 | 0% | 2.4% |
| Magnesium (mg/day) | 199.1 (108.7-289.5) | 212.4 (88.4-561.3) | AI: 300 | 0% | 8.3% |
| Phosphorus (mg/day) | 1021.1 (686.6-1691.7) | 1073.8 (316.5-2183.5) | AI: 550 | 100% | 95.2% |
| Potassium (mg/day) | 2573.9 (1598.7-3643.1) | 2618.7 (1147.4-7356.7) | AI: 3500 | 6.6% | 15.5% |
| Zinc (mg/day) | 8.3 (4.7-11.2) | 8.5 (3.2-19.6) | Range AR: 7.5-11.5 | 66% | 45.2% |
|  |  |  | Range PRI: 9.1-14.3 | 40% | 34.5% |
| Vitamin B12 (mcg/day) | 2.7 (0.9-6.13) | 2.6 (0-7) | AI: 4.5 | 13% | 10.7% |
| Vitamin D (mcg/day) | 1.4 (0.2-4.4) | 1.5 (0.03-7.9) | AI: 15 | 0% | 0% |
| Folate (mcg/day) | 299.1 (134.7-406.9) | 308.1 (106.2-609) | AI: 600 | 0% | 1% |
